# Evolutionary Insights of the ZW Sex Chromosomes in Snakes: A New Chapter Added by the Amazonian Puffing Snakes of the Genus *Spilotes*

**DOI:** 10.3390/genes10040288

**Published:** 2019-04-09

**Authors:** Patrik F. Viana, Tariq Ezaz, Marcelo de Bello Cioffi, Breno Jackson Almeida, Eliana Feldberg

**Affiliations:** 1 Instituto Nacional de Pesquisas da Amazônia, Coordenação de Biodiversidade, Laboratory of Animal Genetics, Av. André Araújo 2936, Petrópolis, Manaus CEP 69067-375, AM, Brazil; feldberg@inpa.gov.br; 2 Institute for Applied Ecology, Faculty of Science and Technology, University of Canberra, Canberra, ACT 2616, Australia; Tariq.Ezaz@canberra.edu.au; 3 Departamento de Genética e Evolução, Universidade Federal de São Carlos, São Carlos 13565-905, SP, Brazil; mbcioffi@ufscar.br; 4 Centro Amazônico de Herpetologia—Amazonian Center for Herpetology, Benevides 68795-000, PA, Brazil; brenojlalmeida@hotmail.com

**Keywords:** advanced snakes, sex chromosomes, heterochromatin, CGH, repeat accumulation, Amazonian snakes

## Abstract

Amazonian puffing snakes (*Spilotes*; Colubridae) are snakes widely distributed in the Neotropical region. However, chromosomal data are scarce in this group and, when available, are only limited to karyotype description using conventional staining. In this paper, we focused on the process of karyotype evolution and trends for sex chromosomes in two Amazonian Puffer Snakes (*S. pulllatus* and *S. sulphureus*). We performed an extensive karyotype characterization using conventional and molecular cytogenetic approaches. The karyotype of *S. sulphureus* (presented here for the first time) exhibits a 2n = 36, similar to that previously described in *S. pullatus*. Both species have highly differentiated ZZ/ZW sex chromosomes, where the W chromosome is highly heterochromatic in *S. pullatus* but euchromatic in *S. sulphureus*. Both W chromosomes are homologous between these species as revealed by cross-species comparative genomic hybridization, even with heterogeneous distributions of several repetitive sequences across their genomes, including on the Z and on the W chromosomes. Our study provides evidence that W chromosomes in these two species have shared ancestry.

## 1. Introduction

The Colubridae family represents one of the most widely distributed groups of Caenophidian snakes, with more than 1800 species currently recognized [1]. It is considered to be a monophyletic group supported by both morphological and molecular analyzes [2,3,4,5,6]. Many species are found in the Neotropical region, which is well known for its high biodiversity and complex evolutionary history [7,8,9,10]. The Amazon puffing snakes (*Spilotes* spp.) are among the largest species of the New World Colubrids [11,12,13], occurring in different morpho-climatic landscapes in South America [14,15,16]. Previously, *Spilotes sulphureus* was described within the *Pseustes* genus [17]. However, Jadin et al. [18] relocated ‘*Pseustes sulphureus*’ to *Spilotes* genus. The renamed *Spilotes sulphureus* became the sister taxon of *S. pullatus* and together formed a monophyletic group that is a sister taxon to the genus *Phrynonax*, all of which together constitute a monophyletic group.

The vast majority of snakes have diploid numbers (2n) of 36 chromosomes, containing 16 macrochromosomes and 20 microchromosomes [19,20]. However, variations involving macro and micro karyotypic structure and a diploid number have also been reported in different families across snake lineages [21,22,23,24,25]. Although different modes and systems of sex determination have been described (e.g.,) in reptiles [26,27,28,29,30], only genetic sex determination (GSD) with female heterogamety (ZZ/ZW system) occurs in snakes [26,31], although an XY system has been proposed for *Boa imperator* and *Python bivittatus* [32]. Nevertheless, the sex chromosomes of many species of snakes remained undifferentiated, with no huge morphological shifts (e.g., Boidae and Phytonidae), with a low degree of differentiation between Z and W sex chromosomes [33,34,35,36], making them undetectable using conventional cytogenetics techniques. Even in Caenophidian snakes, such as in some Colubridae [37], where several repetitive sequences are known to be the main source of differentiation of W sex chromosomes [38], they can remain undetectable. Likewise, the Z chromosome seems to share the same gene content across ancestral and advanced snakes [33,35,39], without large morphological modifications [40].

It is a common feature for some reptile lineages to present a limited level of degeneration of the sex chromosomes, which makes them undetectable under conventional cytogenetic procedures. However, fine-scale molecular cytogenetic techniques (e.g., Comparative Genomic Hybridization; Chromosomal Painting; Bacterial Artificial Chromosome - BAC) have been providing a better understanding regarding the processes of evolution of the sex chromosomes in vertebrates (reviewed in [41]), as well as in comparative approaches among related species [42,43].

So far, most of the refined studies regarding the evolutionary trends of sex chromosomes in snakes were performed only on some Caenophidians´ lineages [34,37,38,44], which represent a gap in the knowledge of the evolutionary pathways of the W across snake lineages. For instance, in the most recently evolved clades of Caenophidians (*Spilotes* and *Phrynonax*), only conventional cytogenetic data exist [22]. For *Spilotes*, the cytogenetic data available are limited to a karyotype description for the two species of the *Spilotes* genus (*S. pulllatus*: 2n = 36 and *S.sulphureus*: 2n = 38) using conventional staining [22]. In this paper, we focused on the process of karyotype evolution and trends of sex chromosome differentiation in the clade that harbors the most recently evolved species among Caenophidian snakes. In order to achieve this, our aim was to perform an extensive karyotype characterization using conventional and molecular cytogenetic approaches in two Amazonian Puffer Snakes (*S. pulllatus* and *S. sulphureus*), to characterize sex chromosome differentiation, as well as to describe cytotaxonomy in the *Spilotes* clade.

## 2. Materials and Methods

### 2.1. Sampling, Mitotic Chromosomes Preparation, C-Banding, and Ag-NORs

The snakes were collected under permission granted by Instituto Chico Mendes de Conservação da Biodiversidade (ICMBio) number: 45275-18 along the Amazon Region. We analyzed 1 male and 1 female of *Spilotes sulphureus* (Far Western and Central Amazon) and 2 males and 2 females of *Spilotes pullatus* (Eastern Amazon). Chromosomal preparations were obtained through in vitro culture of blood [45].

The C-positive heterochromatin and Ag-NORs (nucleolar organizer regions) were detected according to Sumner [46] and Howell and Black [47], respectively.

### 2.2. Probes for Chromosome Hybridization

The 18S rDNA and (TTAGGG)_n_ probes were isolated according to Gross et al. [48] and Ijdo et al. [49], respectively. Both probes were labeled with digoxigenin-11-dUTP using Dig-Nick kit (Bio-NickTranslation Mix, Roche, Mannheim, Germany) following the manufacturer’s recommendations (Roche, Mannheim, Germany). Microsatellites motifs (AC)_15_, (AG)_15_, (ATTC)_8_, (ATCC)_8_, and (GATA)_8_ were used directly labeled with Cy-3 during the synthesis [50].

### 2.3. Fluorescence in Situ Hybridization (FISH) for Repetitive DNA Mapping

Fluorescence in situ hybridization (FISH) followed the protocol described by Pinkel et al. [51], with minor modifications according to Viana et al. [36]. The chromosome slides were denatured in 70% formamide/2 × SSC at 70 °C, and further dehydrated in ethanol series (70%, 85%, and 100%), for 2 min each. In regard to the hybridization mixture, 20 µL (100 ng of each probe, 50% deionized formamide and 10% dextran sulfate) were dropped on the slides, and the hybridization was performed for 24 h at 37 °C in a moist chamber containing distilled water. The chromosomes were counterstained with 4′ 6-diamidino-2-phenylindole (DAPI, 1.2 µg/mL) and mounted in antifade solution (Vector, Burlingame, CA, USA).

### 2.4. Preparation of Probes for Comparative Genome Hybridization (CGH)

The gDNA of males and females of both *Spilotes* species were extracted from blood using the Wizard^®^ Genomic Purification kit (Promega, Madison, WI, USA), according to the manufacturer’s recommendations. Two different experimental designs were used for this study. In the first set of experiments, we focused on intraspecific comparisons, with special emphasis on the molecular composition of the sex chromosomes of *S. pullatus.* In this case, female-derived gDNA of *S. pullatus* was labeled with biotin-16-dUTP and male gDNAs with digoxigenin-11-dUTP by means of nick translation as described above. The final hybridization mixture for each slide (20 μL) was composed of male- and female-derived gDNAs (500 ng each), 20 μg of male-derived C_0_t-1 DNA (i.e., fraction of genomic DNA enriched for highly and moderately repetitive sequences), prepared according to [52], and the hybridization buffer containing 50% formamide, 2 × SSC, 10% SDS, 10% dextran sulfate and Denhardt´s buffer, pH 7.0. In the second set of experiments (interspecific genomic comparisons), the gDNA of female specimens of *S. sulphureus* and *S. pullatus* were hybridized against metaphase chromosomes of female *S. pullatus*. For this purpose, female-derived gDNAs of *S. sulphureus* was labeled with digoxigenin-11-dUTP using DIG-nick-translation Mix (Roche, Mannheim, Germany), while female-derived gDNA of *S. pullatus* was labeled with biotin-16-dUTP using BIO-nick-translation Mix (Roche). The final probe cocktail for each slide was composed of 500 ng of female-derived gDNA of each species, 20 μg of male-derived C_0_t-1 DNA of *S. pullatus*, and 20 μg of male-derived C_0_t-1 DNA of *S. sulphureus*. The probe was ethanol-precipitated, and the dry pellets were resuspended in hybridization buffer, as described above. 

### 2.5. Comparative Genomic Hybridization (CGH)

CGH experiments were performed according to Symonová et al. [53]. The slides were incubated at 37 °C in a dark humid chamber for 72 h. The hybridization signal was detected with anti-digoxigenin-Rhodamin (Roche) diluted in 0.5% bovine serum albumin (BSA) in phosphate-buffered saline solution (PBS), and avidin-FITC (Sigma, St Louis, MO, USA) diluted in PBS containing 10% normal goat serum (NGS). The chromosomes were counterstained with DAPI (1.2 µg/mL) and mounted in an antifade solution (Vector, Burlingame, CA, USA).

### 2.6. Microscopic Analyzes

At least 30 metaphase spreads per individual were analyzed to confirm the 2n, karyotype structure, and FISH results. Images were captured using an Olympus BX51 microscope (Olympus Corporation, Ishikawa, Japan) with CoolSNAP. Chromosomes were classified as macrochromosomes (M) and microchromosomes (mi) or as metacentric (m), submetacentric (sm), subtelocentric (st), and acrocentric (a), according to Levan et al. [54].

## 3. Results

### 3.1. Karyotype

**Figure 1:**
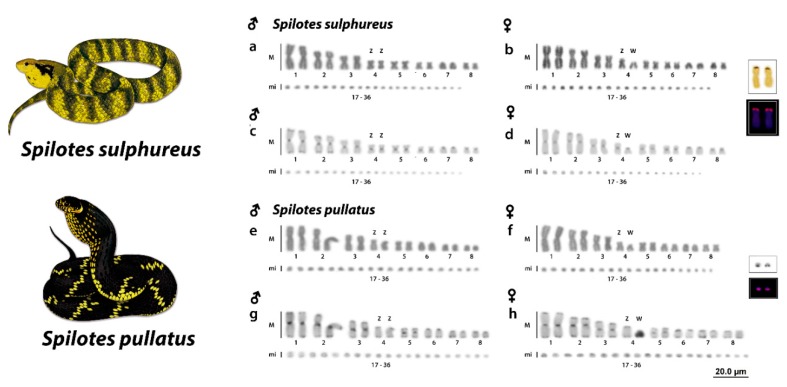
Karyotypes of *Spilotes sulphureus* (**A**,**B**) and *Spilotes pullatus* (**E**,**F**) in Giemsa-staining and C-banding (**C**,**D**,**G**,**H**), respectively, chromosomes showing the heteromorphism of the fourth pair in both females. Note the euchromatic and heterochromatic W chromosomes in *S. sulphureus* and *S. pullatus*, respectively (**D**,**H**). Highlighted in the boxes the mapping of nucleolar organizer regions (NORs) and rDNA 18S. Illustrations by Bruna Borges.

Both species presented a 2n = 36 chromosomes and 16 M and 20 mi. *S. sulphureus* presented 8 m + 4 sm + 2 st + 2 a and 20 mi, with a fundamental number (NF) equal to 50 for males (Figure 1a,c) and 7 m + 4sm + 2st + 3a M and 20 mi, and a fundamental number (NF) equal to 49 for females (Figure 1b,d). *S. pullatus* had 6m + 4sm + 4st + 2a and 20 mi for males (Figure 1e,g) and 6 m + 3 sm + 5 st + 2 a and 20 mi for females (Figure 1f,h). A remarkable heteromorphism was identified in both *Spilotes* species, evidencing the presence of a heteromorphic ZZ/ZW sex chromosome system. The sex chromosomes in both species correspond to the fourth pair. However, Z chromosomes are m and sm in *S. sulphureus* and *S.pullatus*, respectively. In both species, the W chromosomes were highly degenerated and were found to be a and st chromosomes in *S. sulphureus* and *S.pullatus*, respectively. (Figure 1B,F).

### 3.2. Ag-NORs, 18S rDNA, and C-Positive Heterochromatin

Ag-NORs were located on the short arms of one pair of M (second pair) in *S. sulphureus* and in a pair of mi in *S. pullatus*. These sites were confirmed by the mapping of the 18S rDNA sequences (Figure 1, boxed). The C-positive heterochromatin were found in all chromosomes, with a preferential location on the centromeric and telomeric regions in both males and females of the two *Spilotes* species. Interestingly, the W sex chromosome was not completely heterochromatic in *S. sulphureus*, showing only a few centromeric markings (Figure 1D). On the contrary, the W chromosome of *S. pullatus* was entirely heterochromatic (Figure 1H). The microchromosomes showed only spread centromeric bandings (Figure 1C,D,G,H).

### 3.3. Chromosomal Mapping of Microsatellite Motifs and (TTAGGG)n Sequences

**Figure 2:**
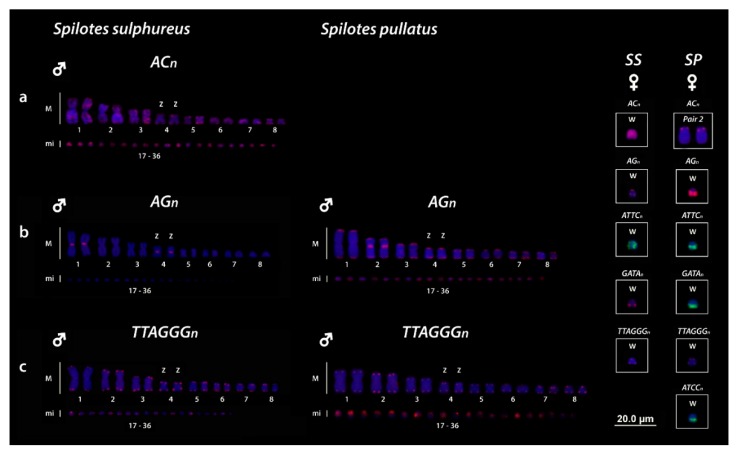
Mapping of (AC)n (**a**), (AG)n (**b**) and (TTAGGG)n (**c**) on the chromosomes of *S. sulphureus* and *S. pullatus*. AC_n_ repeats were evidenced on the second pair of both species and on both Z and W chromosomes of *SS* but absent in the Z and W chromosomes of *SP* (a and highlighted in the boxes). Highlighted in the boxes are the differential accumulation of SSR repeats on the W sex chromosomes for *SS* (*S. sulphureus*) and *SP* (*S. pullatus*).

All five microsatellites repeat motifs used (AC)_15_, (AG)_15_, (ATCC)_8_, (ATTC)_8_, (GATA)_8_ showed hybridization signals on chromosomes of both *Spilotes* species. The (AC)_15_ motifs were hybridized in practically all chromosomes of *S. sulphureus*, including strong signals on the whole W chromosome, whereas *S. pullatus* showed signals only on the short arms of the second pair (Figure 2A, boxed). The (AG)_15_ motifs showed different patterns of hybridization for the two species, being *S. sulphureus* with markings in centromeric position on the first and fourth pairs and telomeric signals in the W chromosome, whereas *S. pullatus* have bitelomeric markings in all chromosomes, with a great accumulation in the centromeric position of the second pair and in the long arms of the W sex chromosome, being only bitelomeric in the Zs (Figure 2B and Highlighted in the boxes). The (ATCC)_8_, (ATTC)_8_ and (GATA)_8_ repeats were hybridized only on the W chromosomes, however, with different patterns for two species. In *S. sulphureus* the (ATTC)_8_ repeats were hybridized on to the whole W, with a diffuse pattern, the (GATA)_8_ showed two markings in the end of long arms, whereas, interestingly, the (ATTC)_8_ repeats were absent in the W (Figure 2, boxed). For *S. pullatus* the (ATCC)_8_ repeats were strongly hybridized only on the long arms of the W chromosome. Both (GATA)_8_ and (ATCC)_8_ repeats showed the same pattern with signals only in the long arms (Figure 2, boxed).

Telomeric repeats (TTAGGG)_5_ revealed terminal hybridization signals in all chromosomes of the complement, without any interstitial sequences (ITSs), even in the W chromosomes for both *Spilotes* species (Figure 2C, boxed).

### 3.4. Comparative Genomic Hybridization (CGH)

**Figure 3:**
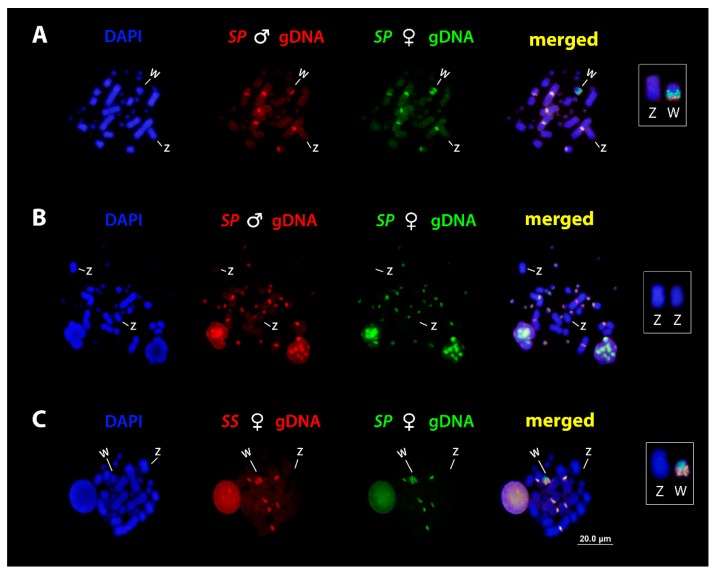
Mitotic chromosome spreads of *S. pullatus* after comparative genomic hybridization (CGH) procedures and cross-Species comparison. Male and female-derived genomic probes from *S. pullatus* mapped against female (**A**) and male (**B**) chromosomes of *S. pullatus*. (**C**) Female-derived genomic probes from *S. pullatus* (*SP*) and *S. sulphureus* (*SS*) mapped against female chromosomes of *S. pullatus*. The common genomic regions are identified as yellow. Please note the shared regions of the W chromosomes in both species and absence of signals on Z chromosome. Z and W sex chromosomes are boxed.

In the first set of experiments, we performed intraspecific comparisons, with special emphasis on the molecular composition of the ZW sex chromosomes of *S. pullatus.* When hybridized to *S. pullatus* female metaphases, the hybridization of the gDNA from both male and female produced intense hybridization signals on macro and micro chromosomes, collocating primarily with C-banded regions. Concerning the sex chromosomes, the merged images revealed that sequences from both sexes are shared on terminal region of the Wq arms, whereas female-specific sequences were concentrated in the pericentromeric region, thus identifying a female-specific region on the W chromosome (Figure 3A). No hybridization signals were observed on the Z chromosome (Figure 3B), which might be due to the low shared content of repetitive sequences on Z. Consecutively, CGH experiments confirmed the pattern.

In the second set of experiments, we performed interspecific hybridization between both species. The results showed that these two species share conserved regions in their chromosomes (Pairs one, two, and seven), as well as with their W sex chromosomes, particularly on the long arms (Figure 3C). No hybridization signals were observed on Z chromosomes.

## 4. Discussion

### 4.1. Cytotaxonomy in Spilotes Clade

Different approaches (e.g., morphological and molecular data) have suggested that *Spilotes*, together with *Phrynonax*, share a most recent common ancestor, and comprise a monophyletic group [2,5,18,55]. They represent the most recently evolved clade of Caenophidian snakes and show a remarkable karyotype diversity ranging from different 2n (36 to 38) and presence and absence or differentiated sex chromosomes [21,22]. In our study, we identified 2n = 36 chromosomes for a *S. sulphureus*, which differs from those found for *S. sulphureus* (= *Pseustes sulphureus*) female by Beçak and Beçak [22]. The karyotype of *S. sulphureus* formerly analyzed (locality of origin mentioned just as Brazil), had 2n = 38 chromosomes with 18 M and 20 mi, which largely diverges from what we found for *S. sulphureus* from the Amazon region under analysis in this paper (2n = 36, 16 M and 20 mi). In addition, we identified a well-differentiated ZW sex chromosome system, absent in *S. sulphureus* analyzed by Beçak and Beçak [22]. Such differences (regarding the 2n and presence/absence of well-differentiated sex chromosomes) certainly calls into question the possibility that two different species were examined. However, given the limited sample sizes of each study, further investigation is warranted. 

Chromosomal data for the *S. pullatus* group were only reported for *S. pullatus anomalepis* (♂ and ♀ from Paraná and São Paulo States/South of Brazil) and *S. pullatus maculatus* (only ♀ from São Paulo State/South of Brazil), variants of the same species [56]. They presented conserved karyotypes with 2n = 36 (16 M and 20 mi) and a ZW sex chromosome system [57], which is similar to those we found for both *Spilotes* species from Amazon region analyzed in this paper. However, the karyotype formulae and morphology of ZW chromosomes largely differ between the species analyzed previously [22,57] and the ones here analyzed (Figure 1). This highlights a notable chromosomal diversity within the *Spilotes* group regarding the chromosomal structure and presence/absence of well-differentiated sex chromosomes.

### 4.2. Ribosomal Sequences in the Evolution of Snakes

The rDNA sequences in Serpentes, more specifically the 18S rDNA, are extremely variable among species of this suborder (e.g., Boidae, Viperidae, and Colubridae families) (see [36]). The presence of single hybridization signals on one pair of microchromosomes may be considered as a plesiomorphic feature for snakes representing ancestral lineage (e.g., Henophidia) [36,58]. On the other hand, hybridization signals on macrochromosomes, on multiple microchromosomes (or even on sex chromosomes) in advanced snakes (Caenophidian) are considered to be derived features [34,59,60,61,62]. This suggests that multiple events of chromosomal rearrangements involving this region may have occurred during karyotype evolution in snakes. 

Although variable among different snake lineages, ribosomal sites seem to show an evolutionary trend in related groups, since, for instance, all Boidae snakes have rDNA located in one pair of microchromosomes [36,58]. However, in snakes from the Colubridae family (from those with known data), the 18S rDNA is usually located in macrochromosomes [34,58], similar to the pattern here identified for *S. sulphureus* (Figure 1).

Unlike several vertebrate species, including Caenophidian snakes, no association of rDNA sequences and the sex chromosome of *S. pullatus* and *S. sulphureus* was identified [34,63,64,65,66]. This likely implies that NORs and rDNA sites located in two microchromosomes of *S. pullatus*, indeed represent an atypical condition for the *Spilotes* clade, as almost all Colubridae, analyzed up to now, present the NORs and 18S rDNA located on a pair of macrochromosomes (often on the 2nd). Perhaps this pattern could be the product of multiple chromosomal rearrangements involving this region and also could represent an ancestral condition for the clade composed by *Spilotes* and *Phrynonax*, once *S. pullatus* (signals on mi) occupy an ancestral position than *S. sulphureus* (signals on M) and *Phrynonax* species.

### 4.3. Trends in the Role of Repetitive Sequences in the W Evolution of Caenophidian Snakes

The accumulation of repetitive sequences, such as rDNA; retrotransposable elements, mini and microsatellites on sex chromosomes has been reported in several vertebrate species (e.g., [44,67,68,69,70,71,72]). Telomere motifs (TTAGGG)n and ITS, for example, have been identified in differentiated sex chromosomes of different groups as mammalian and reptiles [73,74,75] and even in homomorphic sex chromosomes of some Henophidian snakes [36]. In our research, no ITS were identified neither on the ZW sex chromosomes nor in the autosomes of both species analyzed. In contrast, Boid snakes (Henophidian) present multiple ITS in their chromosomes [36], which represents a common feature in several other reptile species (see [76]). This suggests that ITS indeed have an independent amplification throughout Serpentes lineages and are non-necessarily related to chromosomal fusions in all cases.

Microsatellites or simple sequence repeats (SSRs) are short extensions of tandemly repeated DNA sequences and shows several patterns of localization in eukaryotes. The SSRs are involved in the chromosomal structure and even in gene expression [77]. Such motifs have already been identified in sex chromosomes of several reptile species [38,69,78,79] and its general association with sex chromosomes suggests that this feature is one of the likely mechanisms that led to their differentiation [69]. For instance, the (GATA)_n_, component of banded krait minorsatellite (*Bkm*), is a typical microsatellite located in the W chromosomes of Caenophidian [34,38,44,80,81]. However, these sequences are not always confined to the W chromosomes, having been identified also on Y chromosomes in some other lineages, such as in lizards [71], as well as in autosomes of some fish species [82,83]. It has been suggested that *Bkm* motifs play an important role in chromatin organization, being associated with transcription factors and formation of heterochromatin [82,84,85,86,87] and, hence, may have regulatory functions in the process of differentiation of sex chromosomes. The pattern of such W-linked sequences (GATA)_n_ is frequently found in Caenophidian snakes [37,38,40], but interestingly absent in ancestral lineages [88]. However, it is worth highlighting that microsatellites motifs have an evolutionary dynamic origin, differencing among distant groups [69,89], and even at in genera and species level.

SSRs are not exclusive of the sex chromosomes in reptiles, being frequently located also in the autosomes, similar to the scenario here found for (AC)_n_ and (AG)_n_ in both *Spilotes* species. Rovatsos et al. [71], for instance, identified accumulation of the (AC)_n_ motifs in both autosomes and sex chromosomes of *Lialis* species (Pygopodidae). Likewise, we identified (AG)_n_ in both autosomes and sex chromosomes for *S. sulphureus* and a great accumulation in the W of *S. pullatus* (Figure 2). Nevertheless, the females showed a specific amplification for the (AG)_n_ in their W sex chromosomes. Similarly, Matsubara et al. [69] evidenced that (AG)_n_ motif is clearly associated with sex differentiation in a turtle species of the family Chelidae. Thus, the presence of microsatellites motifs in the W chromosomes of phylogenetic divergent groups led us to suppose that these sequences might have some role in the process of the sex chromosome differentiation.

### 4.4. Stability of Gene Content in Snakes Sex Chromosomes

Different approaches using cytogenetic and molecular tools have provided novel information regarding the genetic content and evolution of sex chromosomes in vertebrates (see [29,41]) and independent pathways for the origin of sex chromosomes between reptiles and other vertebrate groups have been proposed [33,34,35,90]. However, sex chromosomes in snakes exhibit relative stability among lineages [33,39].

**Figure 4:**
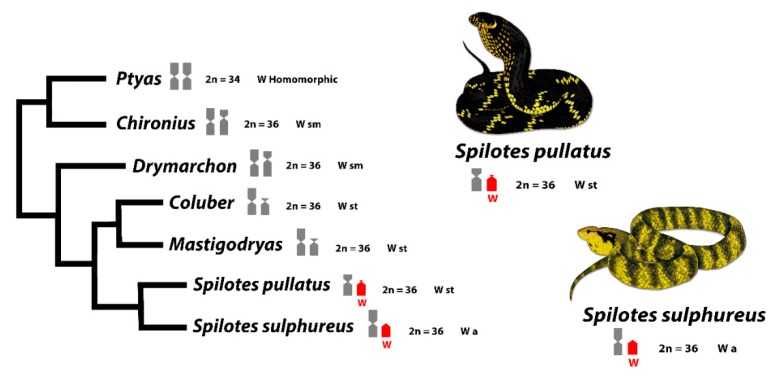
Truncated phylogeny of Caenophidian snakes including *Spilotes* and closely related species showing patterns of sex chromosomes differentiation. The red color highlights the shared W chromosome sequences between species revealed by CGH. Tree topology is adapted from [55]. The sm (submetacentric), st (subtelocentric), and a (acrocentric) corresponds to the W’s morphologies. Illustrations by Bruna Borges.

Although the content of repetitive sequences in the W sex chromosome is stable across Caenophidian species [38], our data, using mapping of several SSRs and C-banding, showed a divergent scenario for the two closely related Colubridae species here analyzed. This could suggest that W chromosome and heterochromatin in Caenophidian snakes are indeed dynamic regarding repetitive sequences content. Moreover, this could explain the high diversity of morphologies of W’s along the evolution of snakes [22,91] and their different stages of evolution with homomorphy, ranging from completely heterochromatic and non-heterochromatic Ws (Figure 4), being the non-heterochromatic W of *S. sulphureus*, a novel finding in Caenophidian snakes.

These findings demonstrate the inherent dynamics of the repetitive DNAs, as well as the pathways that shape the evolutionary history of the sex chromosomes, even among closely related species. This trend for the W chromosome differentiation was already reported for other animal groups, such as fishes [92,93], birds [94], lizards [71], and snakes [38].

Although sharing the same content of some repetitive sequences of the W chromosomes (e.g., AG; ATTC; GATA; Telomere) and with differences in the pattern of accumulation of heterochromatin, the W chromosomes of *S. pullatus* and *S. sulphureus* are very similar, with a subtle divergence in the morphology, being st and a in *S. pullatus* and *S. sulphureus*, respectively. Indeed, it is intriguing to find a non-heterochromatic W chromosome in *S. sulphureus*, once the heterochromatinization and accumulation of *Bkm* sequences in W chromosomes arose in the early Caenophidian snakes [40,88]. Therefore, we can conclude that: (i) The accumulation of *Bkm* repeats, and, consequently, heterochromatinization of W is not a general rule for Caenophidian snakes, or alternatively, (ii) the W chromosomes of *S. sulphureus* represents an exception to this scenario. The real meaning for a non-stability of sex chromosomes in the most advanced clade of Caenophidians could be explained by a possibly recent and independent evolution of sex chromosomes ongoing in *S. sulphureus*. Sex chromosomes can evolve independently between closely related species [27,78,95,96], which seems to be the case of *Spilotes* clade, where the species differ from each other by the SSRs content in both Z and W, presence/absence of well-differentiated sex chromosomes, and euchromatic and heterochromatic Ws. The Z chromosomes, on the other hand, are sm and m in *S. pullatus* and *S. sulphureus*, respectively (Figure 1 and Figure 4), being the Z chromosome from the former, being very similar to the one present in *Ptyas* (Figure 4). These differences could be explained by the rapid and independent evolution of Z chromosomes in relation to the autosomes [97,98], a phenomenon also already seen in the *Ptyas*’ Z chromosomes [99].

Among the phylogenetically related groups to *Spilotes*, an apparent degeneration of the W’s short arms is evident. In *Ptyas*, for instance, both Z and W chromosomes are homomorphic [91,100], whereas the W is sm in *Chironius* and *Drymarchon*, with a similar size to the Z [52]. In *Coluber* and *Mastigodryas*, the W is st, smaller than Z [57,101]. By contrast, degenerated st and a W chromosome are present in *Spilotes* (Figure 4). The *Ptyas’* W chromosome is homomorphic but presents *BKM* repeats only on the W chromosome (like *S. pullatus* and *S. sulphureus*), with no signals in males [37]. Also, specific female sequences (e.g., WAC9, CTNNB1 and CTNNB1W) were identified in this genus [102]. Even being non-stable in its content of sequences, morphology, and different stages of heterochromatinization, the W chromosomes, somehow, have a common evolutionary history among Caenophidian species. Therefore, it seems that the W chromosomes tend to eliminate sequences from the short arms (suggesting that the sex-determining genes are present in the long arms). This scenario opens the opportunity for fine-scale analysis of sex chromosome sequences in this group, which certainly will assist in discovering the mechanisms of ZW sex chromosome evolution in snakes.

### 4.5. Origin and Evolution of Sex Chromosomes in Caenophidian Snakes

**Figure 5:**
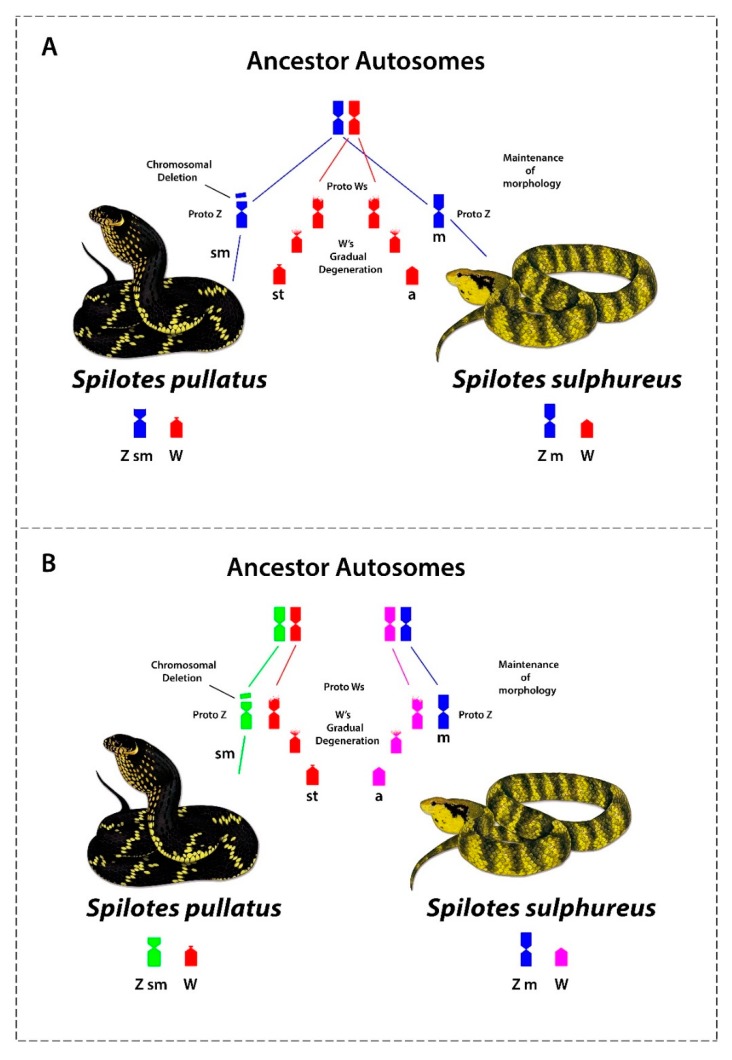
Different possible scenarios of the evolution of the Z and W chromosomes in *Spilotes*. (**A**) Where the Z and W chromosomes in the two *Spilotes* species share ancestry and (**B**) where the *Spilotes* species have evolved their Z and W independently from different autosome pairs. In this scenario, the similarity of sequences revealed by CGH is result of an evolutionary convergent acquisition of repetitive sequences on W.

Comparative cytogenetics revealed that at least 11 genes are shared on the Z chromosomes among ancestral (Henophidian) and advanced snakes (Caenophidian) [33], suggesting an evolutionary conservatism [35,39]. However, our data highlights the evolution and origin of sex chromosomes in our study species. Based on our data we propose two alternative scenarios: Scenario 1. ZW chromosomes of *S. pullatus* and *S. sulphureus* evolved from a common ancestral autosomal pair. The proto Z chromosomes have largely retained its ancestral morphology, except for a segmental deletion of the proximal region of the short arms in *S. pullatus*, while proto W chromosomes have undergone gradual gene loss and degeneration from the short arms, subsequently losing the entire short arms of W chromosomes in *S. pullatus* and *S. sulphureus*. The W chromosomes subsequently accumulated and amplified repetitive sequences, facilitating suppression of recombinations between Z and W chromosomes (Figure 4 and Figure 5a).

Scenario 2. The Z and W chromosomes may have evolved independently from multiple ancestral autosomal pairs. The proto Z and W chromosomes have undergone similar evolutionary changes, as in scenario 1. However, the apparent homologies between the W chromosomes of *S. pullatus* and *S. sulphureus* may likely be result of accumulation and amplification of similar repetitive sequences on the W chromosomes in both species via convergence (Figure 5b).

However, further studies are required to conclusively interpret which of the above scenarios is correct. To conclusively demonstrate homologies between ZW chromosomes between these species, evidence from the sequence level comparison will be required. For instance, this can be achieved by microdissecting Z and W chromosomes and subsequent cross species painting to demonstrate Z chromosomes homologies and sequencing microdissected sex chromosomes to infer synteny.
